# Silencing Attempts of *Bombyx mori* Odorant Receptors Potentially Associated with Oviposition Behavior

**DOI:** 10.3390/insects17030339

**Published:** 2026-03-20

**Authors:** Chanikarn Navakeatpreecha, Piriya Putanyawiwat, Fah Lertkulvanich, Jutarat Jamkratoke, Banthari Chotimanothum, Anchanee Kubera

**Affiliations:** 1 Department of Genetics, Faculty of Science, Kasetsart University, Bangkok 10900, Thailand; 2 The Queen Sirikit Department of Sericulture, Ministry of Agriculture and Cooperatives, Bangkok 10900, Thailand

**Keywords:** silk moth, olfactory receptors, RNAi, gene silencing, egg laying

## Abstract

The silkworm, *Bombyx mori* (*Bm*), is an insect that contributes to industries such as silk production, cosmetics, medicine, and food, as well as to scientific research. A previous study showed that *Bm* odorant receptor (*BmOr*) genes, *BmOr44*, *BmOr54*, and *BmOr63*, may play a major role in oviposition. This research aimed to investigate the function of these three genes using a double-stranded RNA (dsRNA) technique to knock down their expression levels. Our results revealed that the gene-specific dsRNAs could moderately reduce the expression levels of *BmOr44*, *BmOr54*, and *BmOr63* in the silk moth antenna. Silk moths were injected with 50 nM dsRNA*BmOr54* and 100 nM dsRNA*BmOr63* and showed relative oviposition rates under the mulberry leaves condition at 111.45% and 109.58%, respectively, when compared to those with dsRNA*lacZ* injection. The reduction in expression levels of these three genes showed no effect on the oviposition rates of the silk moths without mulberry leaves treatment. The expression levels of these *BmOr* genes were restored after fertilization, suggesting the temporary effects of the dsRNAs. Our findings suggested that variation in *BmOr54* expression level was correlated with changes in oviposition behavior in *Bombyx mori*.

## Simple Summary

We employed dsRNA technique to knock down three *Bombyx mori* odorant receptor genes (*BmOrs*), *BmOr44*, *BmOr54*, and *BmOr63*, to investigate their roles relating to the oviposition behavior of silk moth. Our results revealed that the reduction in the *BmOr54* expression level could increase the oviposition rates of silk moth.

## 1. Introduction

Environmental odorants, including pheromones, are critical mediators of insect behavior and olfactory responses, regulating processes such as mating, oviposition, and predator avoidance [1]. The antennae of insects contain different types of sensilla that play an important role in detecting and odorants [2]. Specific types of odors bind to the corresponding odorant receptors (Ors). Odor perception is mediated by odorant receptors (ORs), which bind specific odor molecules and initiate signal transduction pathways. Previous genomic and functional analyses have identified 170 ORs in the Western honeybee (*Apis mellifera*) [3], 79 ORs in the mosquito (*Anopheles gambiae*) [4], and 62 ORs in the fruit fly (*Drosophila melanogaster*) [5,6,7].

The antennae of insects contain olfactory receptor neurons (ORNs), which express membrane-bound olfactory receptors responsible for odor detection. In insects, olfactory receptors are primarily classified into two major families: odorant receptors (ORs) and ionotropic receptors (IRs). OR proteins typically possess a seven-transmembrane domain topology [8,9,10,11], unlike classical G protein-coupled receptors (GPCRs) [9]. In most neopteran insects, conventional ORs form heteromeric complexes with a highly conserved odorant receptor co-receptor (Orco). In contrast, some basal insects, such as the jumping bristletail, *Machilis hrabei*, possess only a limited number of OR genes and lack an Orco ortholog [10]. The MhOr5 of *M. hrabei* revealed the function of odorant-gated ion channels [11], suggesting that the odors could activate the function of Ors. Various types of odors can interact and trigger the same Or, whereas one single type of odor can stimulate several kinds of Ors [12].

Previous studies have demonstrated that silkworm behavior is strongly influenced by natural chemoattractants. In *Bombyx mori*, male antennae detect bombykal, a major component of the female sex pheromone, thereby triggering courtship behavior [8], whereas female antennae play a critical role in host recognition and oviposition site selection. Sixty-eight Ors were classified in the *B. mori* genome [13]. The expression levels of some *BmOr* genes have been quantified in both female and male adult silk moths [1]. Additionally, some *BmOr* genes exhibit stage-specific expression patterns during development [1]. Tanaka et al. 2009 [1] reported that BmOr56 of *B. mori* silkworms could receive the cis-jasmone compound from mulberry leaves, which affected their behavior. Knockout of the *BmOr56* gene resulted in a complete loss of behavioral attraction to cis-jasmone, confirming its essential role in host-related odor perception [14].

RNA interference (RNAi) is a widely used approach for gene silencing in *B. mori*, enabling the targeted suppression of gene expression through the introduction of double-stranded RNA (dsRNA). In silkworms, dsRNA is commonly delivered by microinjection into embryos, larvae, or pupae. For example, the injection of dsRNA into fifth-instar larvae effectively silenced the expression of the cathepsin L (Cat L)-like cysteine protease gene [15]. Similarly, dsRNA administration during the pupal stage significantly reduced the transcript levels of pheromone gland (PG)-specific genes [16]. In addition to injection-based methods, dsRNA has also been delivered orally through ingestion, providing an alternative strategy for inducing RNAi in silkworms [17,18].

Our previous study [19] indicated that the *BmOr44*, *BmOr54*, and *BmOr63* genes may play major roles in oviposition. We found that the expression levels of *BmOr44*, *BmOr54*, and *BmOr63* in female silkworm moths treated with mulberry leaves during oviposition were significantly lower than those in the control group. Notably, female moths exposed to mulberry leaves during the oviposition period exhibited the highest oviposition rate. In this study, the expression levels of three selected *BmOr* genes were successfully reduced by dsRNA injection during the pupal stage of *B. mori*. Following adult emergence, the oviposition rates of dsRNA-treated female moths were evaluated in the presence of mulberry leaves to assess the functional roles of these receptors in host-associated reproductive behavior.

## 2. Materials and Methods

### 2.1. Moth Treatment Conditions for Oviposition

Silkworm pupae strain J108 were obtained from The Queen Sirikit Department of Sericulture (QSDS), Bangkok, Thailand. This study was done under the certificate of approval for animal care and use for scientific research of Kasetsart University (ACKU65-SCI-031). Silkworms were grown in 6 × 8 m rearing rooms under controlled temperature and relative humidity. The 1st to 3rd instar larvae grew at 25–28 °C and a relative humidity of 80–90%. The 4th to 5th instar larvae were raised at 23–26 °C, with humidity at 70%, while the 5th instar larvae grew at 22–23 °C with humidity below 70% for 6–7 days. The spinning process occurred for 2–3 days until the mature larvae developed into pupae. The cocoons were excised to obtain the 5th day female pupae. The photo period used natural light [20].

### 2.2. dsRNA Preparation and Injection into the B. mori Pupae

**Figure 1:**
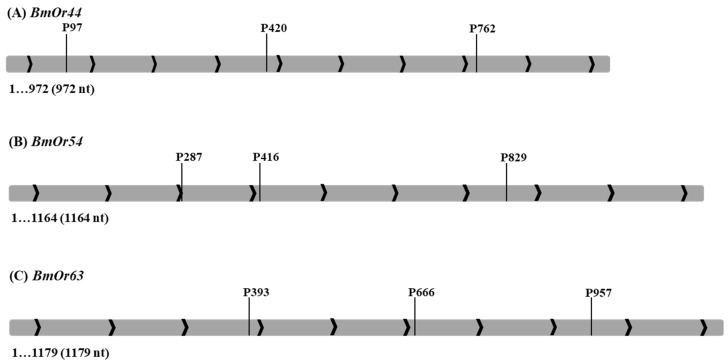
The dsRNAs locations on *BmOr* genes: (**A**) *BmOr44*, (**B**) *BmOr54*, (**C**) *BmOr63*.

The dsRNAs of three *BmOr* genes, *BmOr44*, *BmOr54*, and *BmOr63*, were designed by and purchased from SynBio Technologies (South Brunswick, NJ, USA). The dsRNA sequences are shown in Table 1, and their locations on *BmOr* genes are presented in Figure 1. The dsRNAs were prepared at concentrations of 50 nM and 100 nM. The 5th day female pupae were incubated at 4 °C for 10 min and subsequently injected with 30 µL of dsRNA in the dorsal area. The dsRNA*lacZ* was used as the negative control. The pupae were kept at ambient room temperature (28–30 °C) until they became adult silk moths. Twenty female pupae were used per concentration for each target gene in a single replicate. Three replicates were conducted. Female silk moths were allowed to copulate for 3 h to ensure successful fertilization. Following mating, females were individually transferred to oviposition substrates consisting of a drying filter cloth impregnated with 4% (*w*/*v*) cassava starch adhesive; this setup served as the control condition (no mulberry leaves). In the treatment group, fresh mulberry leaves were placed beneath the filter cloth during the oviposition period [20]. After six hours of oviposition, the female moths were preserved in 95% ethanol and the oviparous eggs and the eggs remaining in the abdomen were counted. The female moths’ antennae, before and after fertilization, were collected for RNA extraction.

### 2.3. Expression Levels of Bombyx mori Odorant Receptor (BmOr) Genes

The expression levels of *BmOr44*, *BmOr54*, and *BmOr63* genes from the antennae of the dsRNA-injected female moths were examined before and after fertilization using different batches of samples. TRIzol^®^ reagent (Thermo Fisher Scientific, Waltham, MA, USA) was used to extract the total RNA from the antennae. Then, cDNA was synthesized from the extracted RNA using the RevertAid First Strand cDNA Synthesis Kit (Thermo Fisher Scientific, Waltham, MA, USA). The cDNA was used as the template for RT-qPCR. The 10 µL reaction mixture of RT-qPCR contained 1 µL of cDNA template, 5 pmol of each primer, and 1x iTaq universal SYBR^®^ green supermix (BIO-RAD, Hercules, CA, USA). The amplification temperature was 95 °C for two min, followed by 35 cycles of 95 °C for 20 s, 55 °C for 15 s, and 72 °C for 20 s. The melting curve analysis was performed at 60–95 °C (0.5 °C increments at 5 s/step). *BmOr* gene-specific primers were used (Table 2). The reference for the transcript quantification was the *actin* gene (Gene ID: 100145915). Ten antennae were used for one replicate at each dsRNA concentration. Three replicates were conducted. The ΔΔC_T_ was determined, and the gene expression levels were calculated by the 2^−ΔΔC^_T_ method using actin as the reference gene and the expression levels of *BmOr* genes in 50 nM dsRNA*lacZ* injection as the control condition. The expression levels of each gene at different dsRNA concentrations were statistically analyzed using *t*-test.

### 2.4. Silk Moth Fertilization and Oviposition

The dsRNA-injected female moths were fertilized at room temperature for 6 h. The male moths were separated, and the females were left at room temperature for 12 h for oviposition, with and without mulberry leaves treatment, under a black cloth covering. The oviparous eggs, and the eggs that remained in the abdomen, were counted. Approximately 20 female moths were used in one replicate for each dsRNA concentration. Three replicates were conducted. The oviposition rates were calculated using the following formula: [oviparous eggs/(oviparous eggs + eggs remaining in the abdomen)] × 100. The oviposition rates were statistically analyzed using one-way ANOVA, and Tukey’s test was used as the post hoc test. The relative oviposition rates were calculated based on the oviposition rates of each condition compared to those of dsRNA*lacZ*-injected silk moths using the following formula: [(100/oviposition rate of dsRNA*lacZ*) × oviposition rate of each condition] − 100. The significant different oviposition rates were examined by *t*-test to obtain the *p*-values. Pearson’s correlation coefficient and the *p*-value were computed to assess the relationship between the positive relative oviposition rate and percentage of gene silencing.

## 3. Results

### 3.1. Expression Levels of Bombyx mori Odorant Receptor (BmOr) Genes Before Fertilization

**Figure 2:**
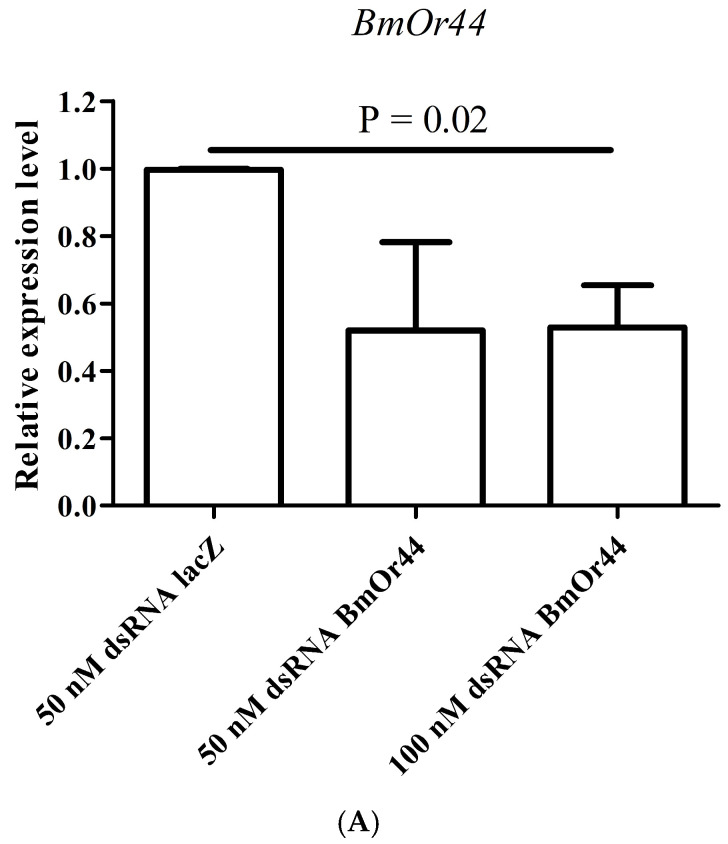

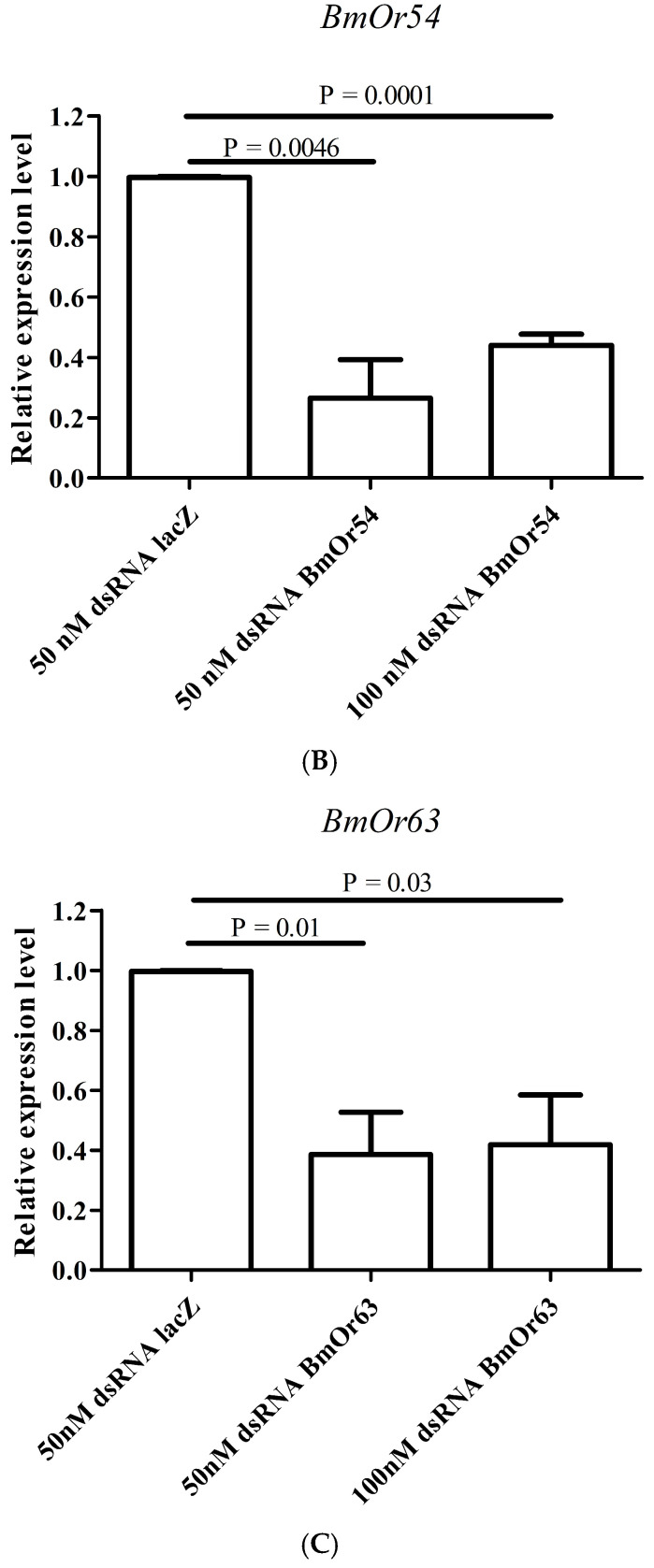
The relative expression levels (mean ± SEM) of *BmOr* genes after dsRNA injection (before fertilization; 3 days post injection): (**A**) *BmOr44*, (**B**) *BmOr54*, (**C**) *BmOr63*.

The relative expression levels of *BmOr44*, *BmOr54*, and *BmOr63* genes in dsRNA-injected female moths’ antennae compared to those of 50 nM dsRNA*lacZ*-injected silk moths before fertilization are shown in Figure 2A–C. The expression levels of all three *BmOr* genes were lower than those of the control 50 nM dsRNA*lacZ* injection. However, the expression level of the *BmOr44* gene after 50 nM dsRNA*BmOr44* injection was not significantly different from the control, whereas other concentrations of dsRNA injection were significantly different. Moreover, the different concentrations of dsRNAs, 50 nM and 100 nM, showed similar relative expression levels in all three *BmOr* genes suggesting the knocking down of these genes in these concentration ranges might be dose-independent. The lowest relative expression level was found in the antennae of silk moths that were injected with 50 nM dsRNA*BmOr54*. The percentage of gene silencing was found to be 35–74%, as shown in Table 3.

### 3.2. Oviposition Rates of dsRNA-Injected Silk Moths

**Figure 3:**
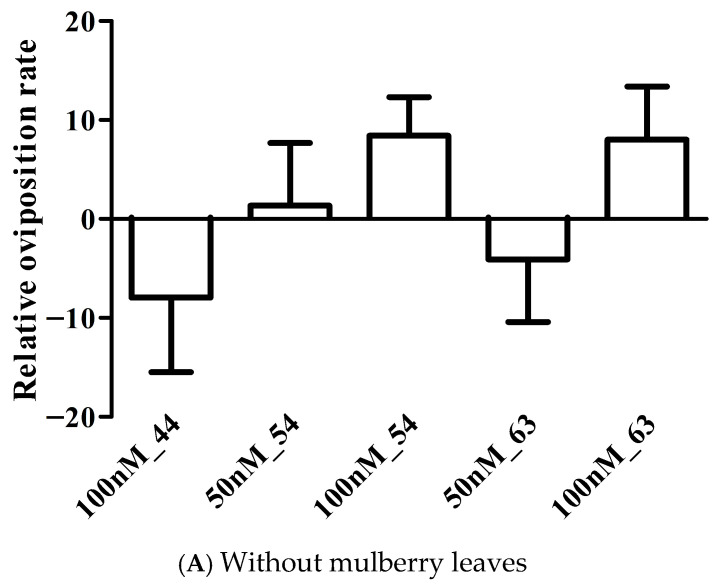

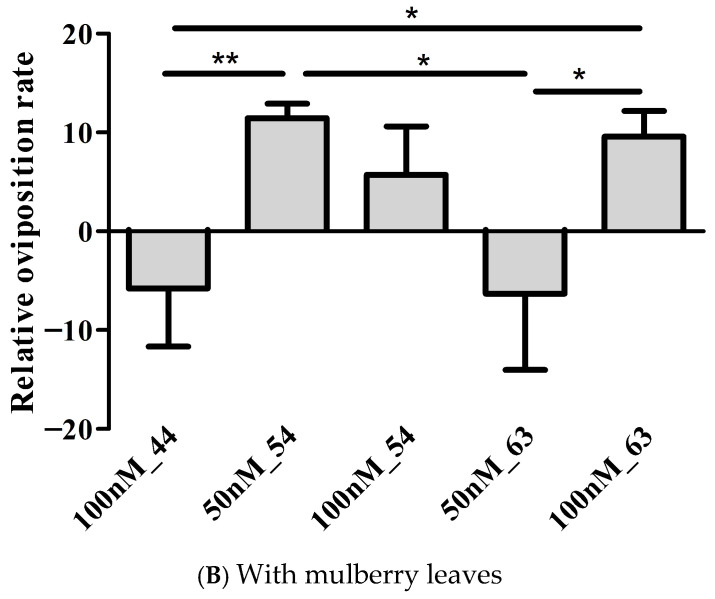
The relative oviposition rates of dsRNA-injected silk moths (mean + SEM). The oviposition rates of each condition compared to those of 50 nM dsRNA*lacZ*-injected silk moths; (**A**) without mulberry leaves condition; (**B**) with mulberry leaves condition; 50 nM and 100 nM represent the dsRNA concentrations, and the number represents the dsRNA specific to target genes. The unpaired Student *t*-test was performed; * = *p* value < 0.05, ** = *p* value < 0.01.

**Figure 4:**
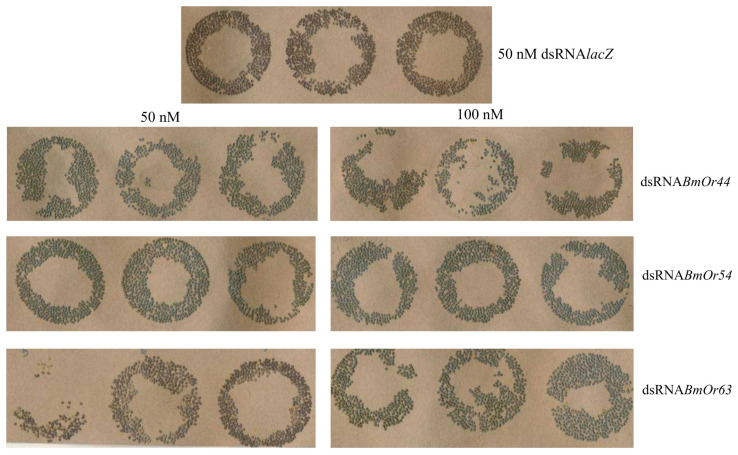
The oviparous eggs on paper after dsRNA injection under mulberry leaves condition.

**Figure 5:**
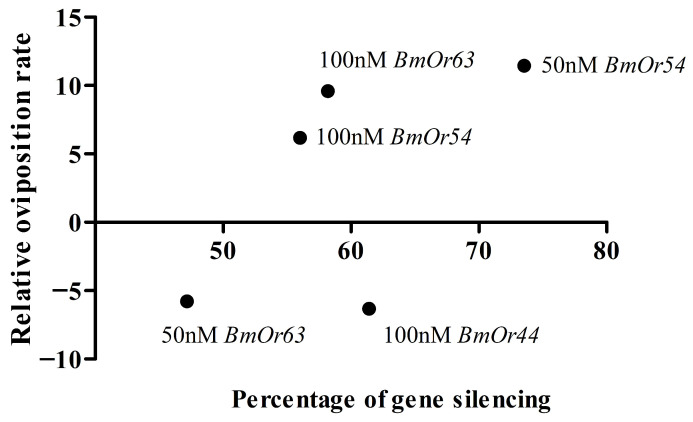
The relationship between relative oviposition rates and percentage of gene silencing. The relative values were calculated compared to those of dsRNA*lacZ*-injected silk moths.

The oviposition rates of dsRNA-injected silk moths are shown in Table 4. The dsRNAs-injected silk moths with 50 nM dsRNA*BmOr54* and 100 nM dsRNA*BmOr63* showed the highest oviposition rates at 94.43% and 92.85%, respectively, and the lowest (79.37%) was found in 50 nM dsRNA*BmOr63*-injected silk moths under the mulberry leaves condition. The oviposition rates of non-dsRNA-injected silk moths without and with mulberry leaves condition were 86.06% and 89.13%, respectively. Under the mulberry leaves treatment, only the injection of 50 nM dsRNA*BmOr54* resulted in a significantly higher oviposition rate, 94.43%, compared with the 50 nM dsRNA*lacZ* control, 84.73%, (*p* < 0.05, *t*-test). Moreover, the oviposition rate of moths injected with 50 nM dsRNA*BmOr54* was significantly higher in the presence of mulberry leaves than in their absence (*p* < 0.05, *t*-test). The relative oviposition rates of dsRNA-injected silk moths were calculated by normalizing each treatment group to the 50 nM dsRNA*lacZ* control. The results obtained in the absence and presence of mulberry leaves are presented in Figure 3A and Figure 3B, respectively. Using one-way ANOVA, there was no significant difference in the relative oviposition rates among the different concentrations of dsRNAs injection without mulberry leaves treatment (F_4,188_ = 1.405, df = 4, *p* value = 0.2340), as shown in Figure 3A. The 100 nM dsRNA*BmOr44* and 50 nM dsRNA*BmOr63* showed a reduction in oviposition rates in both without and with mulberry leaves, whereas other conditions could increase the oviposition rates. The oviparous eggs on paper after dsRNA injection under the mulberry leaves condition are shown in Figure 4. Fifty-nine of 507 dsRNA-injected silk moths (11.64%) showed oviposition rates of less than 50% after fertilization (Supplementary Data). The relationship between the relative oviposition rates and percentage of gene silencing is shown in Figure 5. Pearson’s correlation analysis among the 50 nM dsRNA*BmOr54*, 100 nM dsRNA*BmOr54*, and 100 nM dsRNA*BmOr63* treatments revealed a strong positive correlation; however, this association did not reach statistical significance (r(1) = 0.83, *p* = 0.366).

### 3.3. Expression Levels of Bombyx mori Odorant Receptor (BmOr) Genes After Fertilization

**Figure 6:**
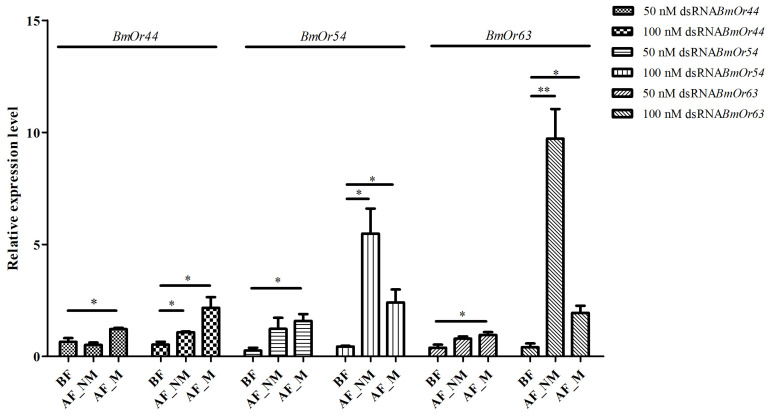
The relative expression levels of *BmOr* genes after dsRNA injection (before and after fertilization) related to those of dsRNA*lacZ*-injected silk moths; BF = before fertilization; AF = after fertilization; NM = no mulberry leaves condition; M = with mulberry leaves condition. The unpaired Student *t*-test was performed; * = *p* value < 0.05, ** = *p* value < 0.01.

The relative expression levels of the *BmOr44*, *BmOr54*, and *BmOr63* genes were also examined in dsRNA-injected female moths after fertilization (five days after dsRNA injection). All expression levels of these three genes, with the exception of *BmOr44* with a 50 nM dsRNA*BmOr44* injection, were restored during fertilization (with and without mulberry leaves treatment), as shown in Figure 6. The restored expression levels of these three genes were not significantly different with and without mulberry leaves during fertilization, with the exception of *BmOr63* after 100 nM dsRNA*BmOr63* injection. These results suggest the temporary effect of gene silencing by dsRNA.

## 4. Discussion

Our previous study found that during the fertilization of silk moths under mulberry leaves treatment, the relative expression levels of *BmOr44*, *BmOr54*, *BmOr56*, and *BmOr63* genes were reduced [19]. These results supported the study of Qiu et al. 2018 [2], which showed that *BmOr44* and *BmOr56* were downregulated in silkworms treated with mulberry leaves. We hypothesized that the disruption of their expression levels might affect the oviposition rate of silk moths. Using the dsRNA technique to reduce the expression levels of specific genes is a powerful method to silence the target genes. In this study, three specific *BmOr* genes, *BmOr44*, *BmOr54*, and *BmOr63*, were selected to knock down to examine their function related to oviposition behavior. The injection of specific dsRNA-*BmOrs* into the *Bm* pupae reduced the expression levels of the corresponding genes in the antennae of female adult moths. However, these three genes were moderately knocked down, as the percentages of gene silencing were 35–74%. Incomplete gene silencing might result in residual mRNA and protein expression, which could preserve partial biological function and attenuate the phenotypic effects. The efficiency of RNAi-mediated knockdown varies, depending on the target sequence, cell type, transfection conditions, and incomplete suppression [21,22]. Therefore, the moderate silencing efficiencies observed here may underestimate the true functional contribution of these genes. Future studies employing multiple independent siRNAs, stable knockdown systems, or CRISPR/Cas9-mediated knockout could help confirm and strengthen these findings. The dsRNA injection at the pupae stage was also employed to silence the *bursicon* gene in silkworm, and its effect was a wing expansion defect [23]. Moreover, the RNA interference (RNAi) technique was introduced to Lepidoptera, with a large number of successful experiments reported [23,24,25,26,27]. In our study, the relative expression levels of each gene obtained at different concentrations—50 nM and 100 nM—of dsRNA injection were found to be similar. The results suggested that the silencing of these genes may be dose-independent. However, RNAi-mediated silencing typically exhibited dose-dependent behavior up to a saturation threshold, beyond which additional dsRNA did not enhance knockdown efficiency [28]. Therefore, the comparable silencing observed at 50 nM and 100 nM may indicate that maximal or near-maximal silencing was already achieved at the lower concentration. Because only two concentrations were tested, definitive conclusions regarding dose dependency cannot be drawn. Further investigation of more concentrations of dsRNAs must be applied to examine this assumption. The oviposition rates of the dsRNA-injected moths revealed a relationship with the expression levels of the three *BmOr* genes. The oviposition rates of dsRNA-injected moths under mulberry leaves, except 100 nM dsRNA*BmOr44* and 50 nM dsRNA*BmOr63*, were found to be higher than those without mulberry leaves. These results strongly supported the findings of our previous study [19], which demonstrated that the mulberry leaves could increase the oviposition rates. Many active compounds have been identified in fresh mulberry leaves, e.g., cis-jasmone, phytene-2, phytol, hexanal, and linalool [1,29]. These compounds could virtually bind to the predicted three-dimensional structures of BmOr44, BmOr54 and BmOr63 [19]. The results suggested specific interactions between the active compounds and the BmOr proteins. It was found that, in silkworm, BmOr56 could strongly interact with cis-jasmone, but that BmOr54 showed weak interaction. Moreover, BmOr63 showed no respond to cis-jasmone [1]. The highest oviposition rate (94.43%) was found in 50 nM dsRNA-*BmOr54*-injected silk moths—the relative expression level of *BmOr54* was the lowest, revealing that the decrease in *BmOr54* could increase the oviposition rates of the silk moth. In addition, the reduction in *BmOr63* gene under 100 nM dsRNA-*BmOr63* injection also increased the oviposition rates. The silencing of *Bactrocera dorsalis odorant binding protein* (BdOBP) gene could also reduce the oviposition rate of the insect [30]. However, after the injection of 100 nM dsRNA*BmOr44* and 50 nM dsRNA*BmOr63*, the oviposition rates of the silk moth were decreased, implying some other factors might affect the oviposition mechanism.

Oviposition behavior in insects is rarely controlled by a single OR but instead arises from the coordinated activity of multiple olfactory receptors. Although BmOr54 was a primary focus of the present study, other receptors—including BmOr56, BmOr44, BmOr63, and potentially additional uncharacterized ORs—may also contribute to host plant recognition and oviposition-site selection. Previous studies have demonstrated that multiple ORs collectively encode attractive and aversive cues, and disruption of individual receptors often shifts behavioral thresholds rather than abolishing oviposition entirely [31]. Functional analyses in *Drosophila melanogaster* further showed that odor perception is mediated by a combinatorial receptor code, in which individual odorants activate specific but overlapping subsets of ORs [7]. In addition, disruption of the *Helicoverpa armigera* OR56 gene in female moths exhibited the loss of their ability to detect oviposition-deterring plant volatiles (ODPs) and consequently lacked oviposition-site preference [32]. This finding provides strong genetic evidence that specific odorant receptors can mediate the perception of deterrent cues that shape reproductive decision-making. In our previous study [19], BmOr54 could virtually bind to the compounds in fresh mulberry leaves: phytol, phytene, and linalool. These compounds had been identified as oviposition deterrents in other insects [33,34]. If BmOr54 participates in detecting inhibitory or modulatory plant volatiles, reducing its expression could attenuate deterrent signaling and shift oviposition behavior toward increased egg laying.

In our study, it was found that 11.64% (59 out of 507) of the dsRNA-injected silk moths showed oviposition rates less than 50% after fertilization, suggesting a drawback of the injection technique. The relative expression levels of *BmOr44*, *BmOr54*, and *BmOr63* were restored after fertilization (5 days post-injection). These results demonstrated the temporary effects of gene silencing by dsRNA. In addition, the restoration of the expression levels of some *BmOr* genes was revealed, suggesting that these genes may be used for other biological pathways.

## Figures and Tables

**Table 1:** The dsRNA sequences of *BmOr* genes.

Gene	dsRNA	Sense (5′–3′)	Antisense (5′–3′)
*BmOr44* ID: 100127045	P97	GAGCAACGAUUUACGUCAAGCTT	GCUUGACGUAAAUCGUUGCUCTT
	P420	CGAUGUUCUUGCGGAAAGACGTT	CGUCUUUCCGCAAGAACAUCGTT
	P762	GAACCAGAACAUAAAGAAAUCTT	GAUUUCUUUAUGUUCUGGUUCTT
*BmOr54* ID: 100144598	P287	GAAUCGUGCUAGUGUUCUAUATT	UAUAGAACACUAGCACGAUUCTT
	P416	GCUGGUGGUUCCUUGCUAAUATT	UAUUAGCAAGGAACCACCAGCTT
	P829	GGUUCAAAUGCACUAACUAUUTT	AAUAGUUAGUGCAUUUGAACCTT
*BmOr63* ID: 100379315	P393	GAUUGAUUAUCAGAGUCAACUTT	AGUUGACUCUGAUAAUCAAUCTT
	P666	GCUUUGUCUGUACUUUCUAUUTT	AAUAGAAAGUACAGACAAAGCTT
	P957	CGUUGAGAGCUCCGAGAUAGCTT	GCUAUCUCGGAGCUCUCAACGTT

**Table 2:** Specific primers for *BmOr* genes and *actin* gene for RT-qPCR.

Gene ID	Name	Sequence (5′–3′)
100145915	*actin*	F: CTCGCCTCCCTCTCTACCTT
		R: CAACAACAACATTCCGTTCG
100127045	*BmOr44*	F: ATGCAGATGGTTTGGATGGC
		R: CAAATTACGCCAAGGACCGT
100144598	*BmOr54*	F: AGTTTGGCTGGGTTCTCGAT
		R: AACCACCAGCTGTTATTGCC
100379315	*BmOr63*	F: ACCCTTACGACACCTCCAAG
		R: TTCAAGTCTTGGCCTAGCGA

F = forward. R = reverse.

**Table 3:** Percentage of gene silencing.

dsRNA	Concentration (nM)	Percentage of Silencing (Mean ± SEM)
*lacZ*	50	0.33 ± 0.33
*BmOr44*	50	34.69 ± 17.05
	100	47.17 ± 12.56
*BmOr54*	50	73.56 ± 12.77
	100	56.00 ± 3.66
*BmOr63*	50	61.42 ± 14.05
	100	58.18 ± 16.58

**Table 4:** Oviposition rates of dsRNA-injected silk moths without and with mulberry leaves (* indicates *p* < 0.05, *t*-test).

dsRNAs	Oviposition Rates (Mean ± SEM)	
	Without Mulberry Leaves	With Mulberry Leaves
50 nM dsRNA*lacZ*	81.63 ± 5.28	84.73 ± 5.36
50 nM dsRNA*BmOr44*	85.66 ± 3.91	88.33 ± 3.68
100 nM dsRNA*BmOr44*	75.15 ± 6.16	79.83 ± 4.98
50 nM dsRNA*BmOr54*	82.73 ± 5.17	94.43 ± 1.24 *
100 nM dsRNA*BmOr54*	88.51 ± 3.18	89.97 ± 4.00
50 nM dsRNA*BmOr63*	78.29 ± 5.15	79.37 ± 6.51
100 nM dsRNA*BmOr63*	88.18 ± 4.38	92.85 ± 2.20

## Data Availability

The data that support the findings of this study are openly available in Zenodo at https://doi.org/10.5281/zenodo.15368610.

## Supplementary Materials

The following supporting information can be downloaded at: https://www.mdpi.com/article/10.3390/insects17030339/s1. The oviposition rates, raw Cq values, relative expression levels, and statistical tests were in the Supplementary Materials.
